# Commentary: Measuring Counterintuitiveness in Supernatural Agent Dream Imagery

**DOI:** 10.3389/fpsyg.2019.02855

**Published:** 2019-12-20

**Authors:** Robert Eugene Sears

**Affiliations:** ^1^Online Department, TCA College, Singapore, Singapore; ^2^School of Intercultural Studies, Fuller Theological Seminary, Pasadena, CA, United States

**Keywords:** dreaming, cognition, counterintuition, supernatural agent concept, CI scheme, complexity drops, unexpectedness, continuity hypothesis

Nordin and Bjälkebring's research on counterintuitiveness (CI) in the dreams of Nepali Hindus is seemingly the first case of applying Barrett's (2008) coding scheme to dream reports. This commentary briefly addresses Nordin and Bjälkebring's main findings with the coding scheme before considering their proposal for the manifestation of supernatural agents (SAs) in dreams. As discussed below, their proposal is vague and ignores other factors that are relevant to oneiric SA manifestation.

## Counterintuitive Objects in Dreams

According to Barrett (2008, 2011), whose work grounds Nordin and Bjälkebring's empirical study, humans naturally develop five general object categories/domains. CI applies to objects with properties that defy basic expectations for its domain (Barrett, 2008, 2011; Purzycki and Willard, 2015). “Talking tree” and “invisible statue that cries” meet CI qualifications because they defy basic expectations for plant and artifact domains. According to Barrett's (2008) coding scheme, these examples have respective CI scores of 1 and 2, given the number of violated expectations per domain.

Using the aforementioned coding scheme on a selection of Hindu supernatural/religious dream reports, Nordin and Bjälkebring report the vast majority of counterintuitive objects in their sample had a CI score of 1. This is consistent with expectations of minimal counterintuitiveness theory concerning cognitive load and narrative transmission (Barrett, 2008; Barrett et al., 2009). Additionally, they found just one counterintuitive object in the majority of dream reports, and counterintuitive agents greatly outnumbered other types of counterintuitive objects in their sample. Altogether, these findings are generally consistent with Barrett et al.'s (2009) study of folktales. The latter employed the CI scheme on an international sample and found artifacts possessing agent properties to be relatively rare. In contrast, artifacts were the most frequent counterintuitive agent in Nordin and Bjälkebring's study. A plausible way of accounting for this difference would be to consider the culture of Nordin and Bjälkebring's subjects. Idols (*murti*) are quite common in the villages and cities of Nepal, and a prominent part of Hindu religious life in particular. Thus, in association with the continuity hypothesis (see e.g., Domhoff, 1996; Bulkeley, 2009), I propose the frequency of counterintuitive object types in a dream report sample is a function of waking circumstances. Hence, a Christian or Muslim sample should reveal a lower frequency of counterintuitive artifacts than a Hindu one.

## Supernatural Agent Cognition

Following McNamara and Bulkeley (2015), Nordin and Bjälkebring argue that the physiology of dreaming entails a diminished sense of personal agency, which could undergird SA cognition in light of the self's corresponding search for extrinsic event causes. Why, however, would subjects interact with SA concepts rather than other non-self concepts during dreaming? Nordin and Bjälkebring argue that certain situations the dreamer faces, viz., those involving perceived threat or anxiety, create inferential demands that make SA concepts attractive because of the latter's association with strategic information. While I would not want to discount their proposal entirely, several important concepts (threat, anxiety, strategic information) are vaguely defined, and other factors related to SA manifestation are ignored.

To support these contentions, I will briefly consider Nordin and Bjälkebring's second dream report example (p. 9). Though Nordin and Bjälkebring's theory applies to dreams rather than dream reports, the latter may nonetheless indicate features of the oneiric experience. Based on the report's details and Nordin and Bjälkebring's generic usage of threat and anxiety terminology, I am unable to conclude that the dreamer experienced these prior to the appearance of SAs. The dreamer's situation is more obviously a case of “unexpectedness” following a domain violation (involving a bowl). The concept of unexpectedness is lacking in Nordin and Bjälkebring's discussion, but it features prominently in several researchers' portrayals of religious/supernatural cognition (e.g., Taves, 2009; Hermans, 2015; Sears, 2016, in press). According to Fortier and Kim (2017), unexpectedness (resulting from drops in algorithmic complexity between expected and actual circumstances) begets agency detection, which leads to SA cognition if naturalistic concepts fail to account. Their “complexity drop model of the supernatural” (CDMS) arguably applies to the example under consideration^1^.

Nordin and Bjälkebring's threat theory and the CDMS explain SA concept activation rather than the visual experience of SAs. The latter is dependent on the former but requires special circumstances. From the perspective of hierarchical predictive coding, sensory data essentially acts as a corrective to mental modeling of perception (Hobson and Friston, 2012; Clark, 2013; Andersen et al., 2014). Based on this perspective, Hobson and Friston (2012) suggest the “bizarre” content of dreams may be due to sensory gating that normally occurs when the body is asleep. Following their reasoning, once SA cognition is activated during dreaming, subjects may continue to envision SAs without their appearance being impeded by sensory information (Sears, in press).

In sum, Nordin and Bjälkebrings's description of oneiric SA cognition is vague and limited. Besides diminished personal agency and threat/anxiety, unexpectedness, and sensory gating/deprivation play important roles in at least some SA dreams. Other factors—such as “ideal” content situations (Sears, 2016)—may likewise be relevant. Interestingly, each of the foregoing factors mentioned with respect to oneiric SA cognition has precedence in treatments of waking SA cognition (cf. Barrett, 2004, 2011; Taves, 2009; Andersen et al., 2014; Fortier and Kim, 2017). This statement is a testament to the principle of continuity between waking and dreaming. Given the typical physiology of dreaming, supernatural cognition and visual SA manifestation may be generally more common during dreaming than waking, though such possibilities stand in need of further research.

## Author Contributions

The author confirms being the sole contributor of this work and has approved it for publication.

### Conflict of Interest

The author declares that the research was conducted in the absence of any commercial or financial relationships that could be construed as a potential conflict of interest.

## Footnotes

^1^Readers of Fortier and Kim's essay will see that they associate complexity drops with domain-general (prototypical) violations rather than the domain-specific (ontological/basic) violations associated with counterintuitiveness in the technical sense (cf. Purzycki and Willard, 2015). They do not defend this division adequately, and I do not think it can be maintained, given that “atypical” objects occasion complexity drops (cf. Dessalles, 2007; Fortier and Kim, 2017, p. 287). Phenomenologically speaking, both types of violations generally create unexpectedness; furthermore, both types of violations have been linked to SA cognition (Fortier and Kim, 2017).
